# Prognostic impact of advanced lung cancer inflammation index and tumor load index in esophageal squamous cell carcinoma after neoadjuvant immunochemotherapy

**DOI:** 10.3389/fimmu.2026.1724061

**Published:** 2026-01-28

**Authors:** Yizhou Huang, Maohui Chen, Zhenyuan Yang, Yongcong Zhang, Chuanquan Lin, Shuliang Zhang, Taidui Zeng, Jun Yu, Chun Chen, Bin Zheng

**Affiliations:** 1 Department of Thoracic Surgery, Fujian Medical University Union Hospital, Fuzhou, China; 2 Key Laboratory of Cardio-Thoracic Surgery (Fujian Medical University), Fujian Province University, Fuzhou, China; 3 National Key Clinical Specialty of Thoracic Surgery, Fuzhou, China; 4 Clinical Research Center for Thoracic Tumors of Fujian Province, Fuzhou, China; 5 Department of Thoracic Surgery, Quanzhou First Hospital, Fujian, China

**Keywords:** advanced lung cancer inflammation index, esophageal squamous cell carcinoma (ESCC), neoadjuvant immunochemotherapy (NICT), survival analysis, tumor load index

## Abstract

**Background:**

Esophageal squamous cell carcinoma (ESCC) carries a high risk of recurrence after neoadjuvant immunochemotherapy and surgery. Both host inflammatory–nutritional status and circulating tumor markers may jointly influence clinical outcomes. We evaluated the prognostic value of the Advanced Lung Cancer Inflammation Index (ALI) and a composite Tumor Load Index (TL) to refine risk stratification in this setting.

**Methods:**

We retrospectively analyzed 460 consecutive ESCC patients who received 2–3 cycles of PD-1 inhibitor plus platinum/taxane-based chemotherapy followed by esophagectomy. ALI was calculated as BMI × albumin/NLR. TL was derived via LASSO Cox regression from pre-treatment SCC-Ag, CEA, and CA19-9. Optimal cutoffs were identified using maximally selected rank statistics (ALI: 31.22; TL: 0.224). Patients were categorized as low-risk (high ALI/low TL), intermediate-risk (high ALI/high TL or low ALI/low TL), and high-risk (low ALI/high TL). Endpoints included overall survival (OS), disease-free survival (DFS), pathologic complete response (pCR), and major pathologic response (MPR).

**Results:**

With a median follow-up of 42 months, 3-year OS rates were 84.7%, 66.0%, and 34.6% for the low-, intermediate-, and high-risk groups, respectively (log-rank P < 0.001). Corresponding 3-year DFS rates were 75.4%, 61.6%, and 29.0%. In multivariable Cox models, intermediate- and high-risk groups had progressively worse OS (adjusted HR = 2.11 and 3.43) and DFS (adjusted HR = 1.64 and 2.62) compared with the low-risk reference (all P < 0.01). High-risk status independently predicted lower odds of achieving MPR (adjusted OR = 0.34, P = 0.002) and pCR (OR = 0.07, P = 0.011). A prognostic nomogram integrating risk group, ASA score, MPR, and ypN status showed strong discrimination (C-index = 0.742) and favorable calibration for 2-, 3-, and 4-year OS, with time-dependent AUCs of 0.759, 0.789, and 0.712.

**Conclusions:**

Pre-treatment ALI and TL jointly provide robust and complementary prognostic information in ESCC patients receiving neoadjuvant immunochemotherapy. Low ALI combined with high TL identifies a biologically aggressive subset with poor pathologic response and inferior survival. Integration of ALI and TL may facilitate risk-adapted perioperative strategies and personalized treatment optimization.

## Introduction

Esophageal cancer remains one of the most lethal malignancies worldwide, characterized by high mortality and poor long-term outcomes (1). In East Asia, esophageal squamous cell carcinoma (ESCC) accounts for more than 90% of cases and is typically diagnosed at a locally advanced stage (2). Multimodal therapy has become the standard of care for locally advanced disease, as surgery alone yields unsatisfactory survival outcomes (3–5). Historically, neoadjuvant chemoradiotherapy has achieved pathologic complete response (pCR) rates of only 30–43%, with postoperative recurrence remaining common (5, 6).

Immune checkpoint inhibitors targeting PD-1 have transformed the therapeutic landscape of advanced ESCC, significantly improving survival when combined with chemotherapy (7, 8). Encouragingly, recent trials have extended these benefits to the neoadjuvant setting. Phase II studies have demonstrated that neoadjuvant chemoimmunotherapy is feasible and associated with higher major pathologic response (MPR) and pCR rates than chemotherapy alone, with approximately 40% of patients achieving pCR and manageable toxicity profiles (9, 10). Retrospective data further suggest that neoadjuvant chemoimmunotherapy may improve survival compared with conventional neoadjuvant regimens (11, 12). Nevertheless, clinical responses to PD-1–based neoadjuvant therapy remain heterogeneous (13, 14), underscoring the urgent need for robust, easily accessible biomarkers to predict therapeutic benefit and guide individualized post-neoadjuvant management.

Systemic inflammation and nutritional status are increasingly recognized as key determinants of cancer progression and treatment outcomes (15, 16). Chronic inflammation promotes tumor growth and immune evasion, whereas cancer-associated malnutrition compromises host immunity and treatment tolerance (17). Composite inflammatory indices, such as the neutrophil-to-lymphocyte ratio (NLR), have shown prognostic relevance in ESCC and other cancers, with elevated NLR reflecting a proinflammatory and immunosuppressive milieu linked to poor survival (17, 18). The Advanced Lung Cancer Inflammation Index (ALI), integrating body mass index (BMI), serum albumin, and NLR, provides a comprehensive measure of nutritional and inflammatory status (19, 20). Although originally developed for lung cancer, ALI has since demonstrated prognostic utility in multiple malignancies. In ESCC, a low ALI—indicating systemic inflammation and nutritional depletion—has been correlated with worse survival outcomes (21). However, its prognostic relevance in the context of neoadjuvant chemoimmunotherapy remains largely unexplored.

Tumor burden is another critical prognostic determinant. Serum tumor markers, including CEA, SCC-Ag, and CA19-9, reflect tumor load and biological aggressiveness. Elevated preoperative levels of CEA and SCC-Ag have been identified as independent predictors of poor prognosis in resectable ESCC (22, 23), while high CA19–9 levels correlate with advanced disease across gastrointestinal malignancies (24). Integrating multiple tumor markers may provide a more comprehensive assessment of tumor burden. For instance, Ma et al. proposed a composite model combining SCC-Ag, CEA, and CA19–9 to predict nodal metastasis, demonstrating that patients classified as high-risk exhibited significantly higher lymph node involvement (25).

Building on this concept, we developed a novel Tumor Load Index (TL) derived from three serum tumor markers (SCC-Ag, CEA, and CA19-9) using least absolute shrinkage and selection operator (LASSO) regression. We evaluated its prognostic value in conjunction with ALI among ESCC patients who underwent neoadjuvant chemoimmunotherapy followed by surgery. We hypothesized that integrating an inflammation–nutrition index (ALI) with a tumor burden index (TL) would enable more precise prediction of pathologic response and long-term survival than either parameter alone.

## Methods

### Study design and ethics

This retrospective cohort study included 460 consecutive patients with locally advanced, clinically non-metastatic (cM0) ESCC who received neoadjuvant chemoimmunotherapy followed by esophagectomy between January 2021 and June 2023 at Fujian Medical University Union Hospital and Quanzhou First Hospital. The study was conducted in accordance with the Declaration of Helsinki and approved by the Ethics Committee of Fujian Medical University Union Hospital (Approval No. 2025KY672). Owing to its retrospective nature, the requirement for written informed consent was waived.

### Eligibility criteria

Patients were eligible if they met all of the following criteria: (1) histologically confirmed, newly diagnosed, locally advanced, and resectable ESCC; (2) receipt of two or three cycles of PD-1 inhibitor plus platinum- and taxane-based chemotherapy as neoadjuvant treatment; and (3) availability of complete clinical, pathologic, and follow-up data. Exclusion criteria included: (1) distant metastasis or unresectable disease identified radiographically or intraoperatively; (2) prior malignancy or anticancer therapy; (3) autoimmune disease, active infection, or other contraindications to immunotherapy; and (4) incomplete baseline or follow-up data precluding analysis.

### Treatment protocol

All patients received two or three cycles of intravenous PD-1 inhibitor combined with platinum- and taxane-based chemotherapy. PD-1 inhibitors included nivolumab, camrelizumab, tislelizumab, sintilimab, or pembrolizumab, administered at standard doses every three weeks. Surgery was performed 4–6 weeks after the final neoadjuvant cycle. Transthoracic esophagectomy with systematic lymphadenectomy was conducted, and pathologic staging followed the 8th edition of the American Joint Committee on Cancer (AJCC) TNM classification.

### Calculation of ALI and tumor load index

Baseline demographic and laboratory data were collected prior to neoadjuvant therapy. ALI was calculated as BMI × serum albumin (g/L)/NLR, where NLR was defined as the ratio of absolute neutrophil count to lymphocyte count. A higher ALI reflects a favorable nutritional and inflammatory profile, whereas a lower ALI indicates malnutrition and systemic inflammation.

To quantify tumor burden, five serum biomarkers (SCC-Ag, CYFRA21-1, CEA, CA19-9, and CA125) were initially considered. A LASSO Cox regression model was used for variable selection, with overall survival as the dependent variable. The optimal penalty parameter (λ) was determined via 10-fold cross-validation to minimize partial likelihood deviance. At the optimal λ, three variables—SCC-Ag, CEA, and CA19-9—retained nonzero coefficients and were incorporated into a weighted linear formula to generate the TL: TL = 0.0228 × SCC-Ag (ng/mL) + 0.0278 × CEA (ng/mL) + 0.0027 × CA19-9 (U/mL). This composite index quantitatively reflects overall tumor biomarker burden, with higher weights for CEA and SCC-Ag, suggesting stronger associations with disease aggressiveness.

### Cutoff determination and grouping

Optimal cutoff values for ALI and TL with respect to overall survival were identified using maximally selected rank statistics, a survival tree–based method that determines thresholds maximizing the log-rank statistic. The optimal pretreatment cutoffs were 31.22 for ALI and 0.224 for TL. Patients were categorized into high-ALI and low-ALI groups, and high-TL and low-TL groups, respectively. Four subgroups were subsequently defined based on combined ALI/TL status to evaluate the interaction between nutritional–inflammatory condition and tumor burden in predicting treatment benefit.

### Pathologic response evaluation

Pathologic response to neoadjuvant therapy was assessed independently by two gastrointestinal pathologists blinded to clinical data. Tumor regression was graded according to ypT category and tumor regression grade (TRG). pCR was defined as the absence of residual viable tumor cells in both the primary site and lymph nodes (ypT0N0). MPR was defined as ≤10% residual viable tumor cells within the primary tumor bed. Post-neoadjuvant stage (ypT0–4, ypN0–3) and achievement of MPR or pCR were recorded for all patients.

### Follow-up and endpoints

Postoperatively, patients were followed every 3 months during the first 2 years and every 6 months thereafter. Follow-up assessments included physical examination, thoracoabdominal CT, and endoscopy when indicated. OS was defined as the interval from surgery to death from any cause, and DFS as the interval from surgery to the first recurrence or death. The median follow-up duration was 42 months, with data updated as of September 30, 2025.

### Statistical analysis

Continuous variables were presented as mean ± standard deviation (SD) or median (range) and compared using the Student’s t-test or Mann–Whitney U test as appropriate. Categorical variables were analyzed with the chi-square or Fisher’s exact test. Multivariable logistic regression was used to identify independent predictors of MPR, with odds ratios (ORs) and 95% confidence intervals (CIs) reported.

Survival was estimated using the Kaplan–Meier method and compared by log-rank test. Cox proportional hazards models were applied to estimate hazard ratios (HRs) for OS and DFS. Variables with P < 0.05 in univariate analyses were entered into multivariable modeling.

A prognostic nomogram was constructed based on the final multivariable Cox model to predict 2-, 3-, and 4-year OS probabilities. Model discrimination was assessed using Harrell’s concordance index (C-index) and time-dependent receiver operating characteristic (ROC) curves. Calibration was evaluated with 1,000 bootstrap resamples to produce bias-corrected calibration plots. All analyses were performed using R software (version 4.3.1), and a two-sided P < 0.05 was considered statistically significant.

## Results

### Survival outcomes by ALI and TL

**Figure 1:**
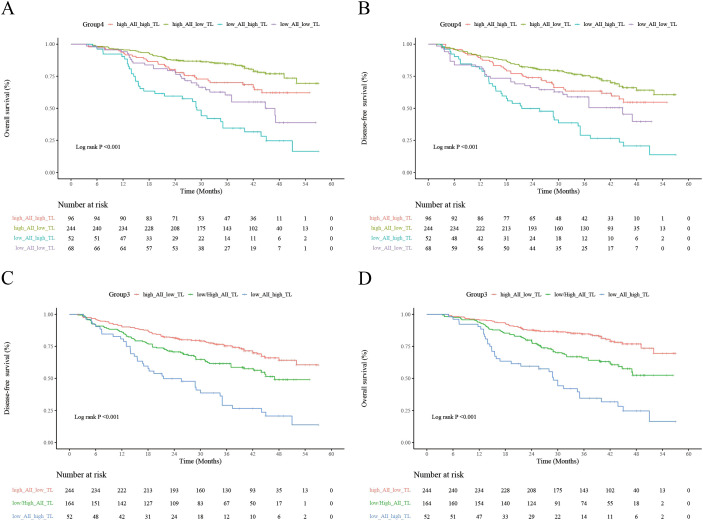
Prognostic impact of ALI and TL on survival in ESCC patients receiving neoadjuvant immunochemotherapy. **(A, B)** Kaplan–Meier curves for overall survival and disease-free survival according to the four-category classification (ALI-high/TL-low, ALI-high/TL-high, ALI-low/TL-low, and ALI-low/TL-high). **(C, D)** Kaplan–Meier curves for overall survival and disease-free survival based on the simplified three-tier risk stratification (low-, intermediate-, and high-risk groups derived from combined ALI–TL status).

With a median follow-up of 42 months, overall and disease-free survival for the entire cohort are shown in **Supplementary Figure 1**. Kaplan–Meier analyses stratified by combined ALI/TL status (**Figures 1A, B**) revealed clearly distinct survival trajectories across the four predefined subgroups. Patients with high ALI/low TL achieved the most favorable outcomes, whereas those with low ALI/high TL experienced the poorest prognosis. The remaining two subgroups—high ALI/high TL and low ALI/low TL—exhibited intermediate outcomes with largely overlapping survival curves, and their difference was not statistically significant (OS, P = 0.14), indicating broadly comparable risks.

Based on these findings, a simplified three-tier classification was derived. Kaplan–Meier curves using this model (**Figures 1C, D**) showed well-separated survival patterns. The 3-year OS and DFS rates were 84.7% and 75.4% for the low-risk group, 66.0% and 61.6% for the intermediate-risk group, and 34.6% and 29.0% for the high-risk group, respectively. Both OS and DFS differed significantly among the three strata, with a highly significant log-rank trend (P < 0.001).

On univariate Cox regression, both ALI and TL were strong individual prognostic indicators. Low ALI was associated with increased risks of death (HR = 2.98, P < 0.001) and recurrence (HR = 2.37, P < 0.001) compared with high ALI. Similarly, high TL predicted poorer OS (HR = 2.12, P < 0.001) and DFS (HR = 1.76, P < 0.001). When modeled jointly, the combined ALI/TL grouping provided an even stronger prognostic stratification (**Supplementary Table 1**). ROC analysis (**Supplementary Figure 2**) showed that the combined ALI/TL model (AUC = 0.70) consistently outperformed its individual components (ALI: AUC = 0.66; TL: AUC = 0.61). Additionally, integrating ALI/TL with ypTNM staging enhanced predictive value, increasing the AUC to 0.78 (C-index: 0.73).

### Patient characteristics by ALI and TL

Baseline clinicopathologic characteristics were generally well balanced across the three ALI/TL-defined risk strata (**Table 1**). No significant differences were observed in age distribution, sex, or comorbidity burden (all P > 0.05). As expected from the derivation of the indices, patients with high ALI displayed higher BMI and serum albumin levels and lower neutrophil counts (resulting in lower NLR), whereas those with high TL had markedly elevated circulating SCC-Ag, CEA, and CA19–9 levels (all P < 0.001).

**Table 1:** Baseline clinicopathologic characteristics and laboratory findings across the three ALI–TL risk groups.

Variables	Total (n = 460)	H–L (n = 244)	H–H/L–L (n = 164)	L–H (n = 52)	P
Age, n(%)					0.493
>60	237 (51.52)	130 (53.28)	84 (51.22)	23 (44.23)	
≤ 60	223 (48.48)	114 (46.72)	80 (48.78)	29 (55.77)	
Gender, n(%)					0.142
Male	357 (77.61)	181 (74.18)	132 (80.49)	44 (84.62)	
Female	103 (22.39)	63 (25.82)	32 (19.51)	8 (15.38)	
Hypertension, n(%)					0.726
No	419 (91.09)	222 (90.98)	151 (92.07)	46 (88.46)	
Yes	41 (8.91)	22 (9.02)	13 (7.93)	6 (11.54)	
Diabetes, n(%)					0.479
No	437 (95.00)	231 (94.67)	158 (96.34)	48 (92.31)	
Yes	23 (5.00)	13 (5.33)	6 (3.66)	4 (7.69)	
Tumour location, n(%)					0.053
Upper	71 (15.43)	46 (18.85)	15 (9.15)	10 (19.23)	
Middle	270 (58.70)	142 (58.20)	102 (62.20)	26 (50.00)	
Lower	119 (25.87)	56 (22.95)	47 (28.66)	16 (30.77)	
cT stage, n(%)					0.235
1	12 (2.61)	8 (3.28)	3 (1.83)	1 (1.92)	
2	99 (21.52)	59 (24.18)	30 (18.29)	10 (19.23)	
3	238 (51.74)	130 (53.28)	83 (50.61)	25 (48.08)	
4	111 (24.13)	47 (19.26)	48 (29.27)	16 (30.77)	
cN stage, n(%)					0.266
0	85 (18.48)	43 (17.62)	37 (22.56)	5 (9.62)	
1	124 (26.96)	64 (26.23)	45 (27.44)	15 (28.85)	
2	182 (39.57)	99 (40.57)	63 (38.41)	20 (38.46)	
3	69 (15.00)	38 (15.57)	19 (11.59)	12 (23.08)	
cTNM stage, n(%)					0.110
II	85 (18.48)	45 (18.44)	6 (11.54)	34 (20.73)	
III	219 (47.61)	127 (52.05)	23 (44.23)	69 (42.07)	
IVA	156 (33.91)	72 (29.51)	23 (44.23)	61 (37.20)	
Indicators before neoadjuvant therapy					
Absolute neutrophil count, Mean ± SD	4.22 ± 1.66	3.76 ± 1.19	4.67 ± 2.08	5.00 ± 1.36	<.001
Absolute lymphocyte count, Mean ± SD	1.94 ± 0.60	2.08 ± 0.58	1.86 ± 0.59	1.50 ± 0.46	<.001
Neutrophils/Lymphocytes, Mean ± SD	2.35 ± 1.11	1.86 ± 0.53	2.73 ± 1.40	3.43 ± 0.77	<.001
Albumin, Mean ± SD	40.83 ± 3.91	41.57 ± 3.88	40.49 ± 3.78	38.39 ± 3.33	<.001
BMI, Mean ± SD	21.89 ± 2.96	22.55 ± 3.00	21.41 ± 2.78	20.30 ± 2.45	<.001

H–L, high ALI/low TL (low-risk); H–H/L–L, high ALI/high TL or low ALI/low TL (intermediate-risk); L–H, low ALI/high TL (high-risk).

### Postoperative pathologic findings and complications

Postoperative pathologic outcomes varied significantly among ALI/TL risk groups (**Table 2**). The pCR rate was 20.9% in the low-risk group but only 1.9% in the high-risk group (P = 0.004). Notably, only 1 of 52 high-risk patients achieved pCR, compared with approximately one in five patients in the other two groups. Similarly, MPR rates differed significantly across groups (P = 0.006): 49.2% in the low-risk, 43.3% in the intermediate-risk, and 25.0% in the high-risk group. Postoperative complication rates were comparable among the three strata, with no meaningful between-group differences in overall morbidity.

**Table 2:** Postoperative pathological findings and perioperative outcomes among the three ALI–TL risk groups.

Variables	Total (n = 460)	H–L (n = 244)	H–H/L–L (n = 164)	L–H (n = 52)	P
PCR, n(%)					0.004
No	374 (81.30)	193 (79.10)	130 (79.27)	51 (98.08)	
Yes	86 (18.70)	51 (20.90)	34 (20.73)	1 (1.92)	
MPR, n(%)					0.006
No	256 (55.65)	124 (50.82)	93 (56.71)	39 (75.00)	
Yes	204 (44.35)	120 (49.18)	71 (43.29)	13 (25.00)	
ypT stage, n(%)					0.002
0	96 (20.87)	57 (23.36)	37 (22.56)	2 (3.85)	
1	55 (11.96)	29 (11.89)	18 (10.98)	8 (15.38)	
2	82 (17.83)	45 (18.44)	31 (18.90)	6 (11.54)	
3	212 (46.09)	108 (44.26)	74 (45.12)	30 (57.69)	
4	15 (3.26)	5 (2.05)	4 (2.44)	6 (11.54)	
ypN stage, n(%)					0.009
0	241 (52.39)	131 (53.69)	94 (57.32)	16 (30.77)	
1	123 (26.74)	68 (27.87)	38 (23.17)	17 (32.69)	
2	76 (16.52)	38 (15.57)	22 (13.41)	16 (30.77)	
3	20 (4.35)	7 (2.87)	10 (6.10)	3 (5.77)	
ypT, n(%)					0.009
T 0-2	233 (50.65)	131 (53.69)	86 (52.44)	16 (30.77)	
T 3-4	227 (49.35)	113 (46.31)	78 (47.56)	36 (69.23)	
ypN, n(%)					0.003
ypN-	241 (52.39)	131 (53.69)	94 (57.32)	16 (30.77)	
ypN+	219 (47.61)	113 (46.31)	70 (42.68)	36 (69.23)	
G, n(%)					0.156
G1	140 (30.43)	80 (32.79)	50 (30.49)	10 (19.23)	
G2-3	320 (69.57)	164 (67.21)	114 (69.51)	42 (80.77)	
Adjuant, n(%)					0.079
No	190 (41.30)	97 (39.75)	64 (39.02)	29 (55.77)	
Yes	270 (58.70)	147 (60.25)	100 (60.98)	23 (44.23)	
Pneumonia, n(%)					0.403
No	314 (68.26)	168 (68.85)	107 (65.24)	39 (75.00)	
Yes	146 (31.74)	76 (31.15)	57 (34.76)	13 (25.00)	
Anastomotic leak, n(%)					0.823
No	409 (88.91)	219 (89.75)	144 (87.80)	46 (88.46)	
Yes	51 (11.09)	25 (10.25)	20 (12.20)	6 (11.54)	
Pleural effusion, n(%)					0.312
No	429 (93.26)	228 (93.44)	155 (94.51)	46 (88.46)	
Yes	31 (6.74)	16 (6.56)	9 (5.49)	6 (11.54)	
Arrhythmology, n(%)					0.631
No	433 (94.13)	232 (95.08)	153 (93.29)	48 (92.31)	
Yes	27 (5.87)	12 (4.92)	11 (6.71)	4 (7.69)	
Chylous fistula, n(%)					0.198
No	455 (98.91)	243 (99.59)	160 (97.56)	52 (100.00)	
Yes	5 (1.09)	1 (0.41)	4 (2.44)	0 (0.00)	
Incision infection, n(%)					0.160
No	454 (98.70)	243 (99.59)	160 (97.56)	51 (98.08)	
Yes	6 (1.30)	1 (0.41)	4 (2.44)	1 (1.92)	

H–L, high ALI/low TL (low-risk); H–H/L–L, high ALI/high TL or low ALI/low TL (intermediate-risk); L–H, low ALI/high TL (high-risk); pCR, pathologic complete response; MPR, major pathologic response; G, histological grade.

### Multivariate analysis of pathologic response predictors

After adjustment for baseline clinicopathologic covariates, the ALI/TL risk classification remained an independent predictor of histopathologic response. In univariate analysis, the high-risk phenotype (low ALI/high TL) was associated with markedly lower odds of achieving MPR relative to the low-risk reference (OR = 0.34, P = 0.002), whereas the intermediate-risk group showed a non-significant trend toward reduced response (OR = 0.79, P = 0.243; **Table 3**).

**Table 3:** Univariable and multivariable logistic regression analyses of factors associated with major pathologic response and pathologic complete response.

Variables	MRP				PCR			
	Univariate analysis	P	Multivariate analysis	P	Univariate analysis	P	Multivariate analysis	P
	OR (95%CI)		OR (95%CI)		OR (95%CI)		OR (95%CI)	
Group								
H-L	1.00 (Reference)		1.00 (Reference)		1.00 (Reference)		1.00 (Reference)	
H–H/L–L	0.79 (0.53 ~ 1.17)	0.243	0.77 (0.51 ~ 1.16)	0.215	0.99 (0.61 ~ 1.61)	0.967	0.96 (0.59 ~ 1.58)	0.877
L-H	0.34 (0.18 ~ 0.68)	0.002	0.34 (0.17 ~ 0.68)	0.002	0.07 (0.01 ~ 0.55)	0.011	0.07 (0.01 ~ 0.56)	0.011
Age								
>60	1.00 (Reference)				1.00 (Reference)			
≤ 60	1.16 (0.80 ~ 1.67)	0.441			0.96 (0.60 ~ 1.54)	0.869		
Gender								
Male	1.00 (Reference)				1.00 (Reference)			
Female	0.97 (0.62 ~ 1.50)	0.879			0.83 (0.46 ~ 1.48)	0.518		
Smoking								
No	1.00 (Reference)				1.00 (Reference)			
Yes	1.00 (0.69 ~ 1.45)	0.988			1.10 (0.68 ~ 1.77)	0.691		
Drinking								
No	1.00 (Reference)				1.00 (Reference)			
Yes	1.04 (0.72 ~ 1.50)	0.833			1.33 (0.83 ~ 2.14)	0.237		
Tumour location								
Upper	1.00 (Reference)				1.00 (Reference)			
Middle	1.06 (0.63 ~ 1.80)	0.826			0.84 (0.43 ~ 1.63)	0.598		
Lower	1.21 (0.67 ~ 2.20)	0.520			1.14 (0.55 ~ 2.36)	0.728		
cT stage								
1	1.00 (Reference)		1.00 (Reference)		1.00 (Reference)			
2	0.17 (0.04 ~ 0.80)	0.025	0.15 (0.03 ~ 0.72)	0.018	0.47 (0.14 ~ 1.55)	0.214		
3	0.10 (0.02 ~ 0.46)	0.003	0.09 (0.02 ~ 0.42)	0.002	0.33 (0.11 ~ 1.04)	0.059		
4	0.15 (0.03 ~ 0.71)	0.017	0.14 (0.03 ~ 0.67)	0.014	0.50 (0.15 ~ 1.62)	0.246		
cN stage								
0	1.00 (Reference)		1.00 (Reference)		1.00 (Reference)		1.00 (Reference)	
1	0.55 (0.31 ~ 0.95)	0.034	0.57 (0.32 ~ 1.01)	0.055	0.44 (0.22 ~ 0.85)	0.014	0.46 (0.24 ~ 0.91)	0.025
2	0.54 (0.32 ~ 0.91)	0.021	0.50 (0.29 ~ 0.85)	0.010	0.36 (0.19 ~ 0.68)	0.001	0.38 (0.20 ~ 0.71)	0.002
3	0.79 (0.42 ~ 1.48)	0.457	0.71 (0.37 ~ 1.39)	0.321	0.63 (0.30 ~ 1.31)	0.218	0.72 (0.34 ~ 1.54)	0.402

In the multivariable logistic regression model, the high-risk group retained its strong negative association with MPR (adjusted OR = 0.34, 95% CI 0.17–0.68, P = 0.002), corresponding to an approximately 66% lower likelihood of major pathologic response compared with the low-risk group. The intermediate-risk group remained non-significant (adjusted OR = 0.77, P = 0.215), suggesting that the preservation of either a favorable nutritional–inflammatory profile or a lower tumor marker burden largely maintains treatment sensitivity.

Findings for pCR were concordant: the high-risk group exhibited a markedly reduced probability of pCR (OR = 0.07, P = 0.011), while the intermediate-risk group again showed no significant difference from the low-risk reference (OR = 0.96, P = 0.877).

### Multivariate analysis of survival outcomes

In multivariate Cox regression adjusting for baseline ASA score and adjuvant therapy status, the three-level ALI/TL classification remained an independent prognostic determinant for both OS and DFS. Compared with the low-risk reference, patients in the intermediate-risk group (one unfavorable factor) had more than a twofold increased hazard of death (adjusted HR = 2.11, 95% CI 1.43–3.08, P < 0.001), while those in the high-risk group (both unfavorable factors) had the greatest mortality risk (adjusted HR = 3.43, 95% CI 2.18–5.39, P < 0.001; **Table 4**).

**Table 4:** Univariable and multivariable Cox proportional hazards analyses for overall survival and disease-free survival.

Variables	OS				DFS			
	Univariate analysis	P	Multivariate analysis	P	Univariate analysis	P	Multivariate analysis	P
	HR (95%CI)		HR (95%CI)		HR (95%CI)		HR (95%CI)	
Group								
H-L	1.00 (Reference)		1.00 (Reference)		1.00 (Reference)		1.00 (Reference)	
H–H/L–L	2.19 (1.50 ~ 3.20)	<.001	2.10 (1.43 ~ 3.08)	<.001	1.67 (1.20 ~ 2.33)	0.002	1.64 (1.17 ~ 2.29)	0.004
L-H	5.05 (3.27 ~ 7.79)	<.001	3.43 (2.18 ~ 5.39)	<.001	3.60 (2.43 ~ 5.33)	<.001	2.62 (1.74 ~ 3.95)	<.001
ASA								
I	1.00 (Reference)		1.00 (Reference)		1.00 (Reference)		1.00 (Reference)	
II	1.57 (0.99 ~ 2.51)	0.057	1.50 (0.94 ~ 2.40)	0.090	1.61 (1.07 ~ 2.43)	0.024	1.47 (0.97 ~ 2.23)	0.071
III	2.03 (1.18 ~ 3.48)	0.010	1.76 (1.01 ~ 3.08)	0.046	1.64 (1.00 ~ 2.69)	0.052	1.42 (0.85 ~ 2.38)	0.177
Adjuant								
No	1.00 (Reference)				1.00 (Reference)			
Yes	0.85 (0.61 ~ 1.19)	0.349			1.09 (0.81 ~ 1.47)	0.577		
Age								
>60	1.00 (Reference)				1.00 (Reference)			
≤ 60	1.06 (0.77 ~ 1.47)	0.715			1.03 (0.77 ~ 1.38)	0.864		
Gender								
Male	1.00 (Reference)				1.00 (Reference)			
Female	0.78 (0.52 ~ 1.18)	0.244			0.94 (0.66 ~ 1.35)	0.753		
Hypertension								
No	1.00 (Reference)				1.00 (Reference)			
Yes	0.88 (0.47 ~ 1.62)	0.678			1.05 (0.63 ~ 1.75)	0.859		
Diabetes								
No	1.00 (Reference)				1.00 (Reference)			
Yes	0.72 (0.32 ~ 1.63)	0.433			0.76 (0.37 ~ 1.54)	0.449		
Tumour location								
Upper	1.00 (Reference)				1.00 (Reference)			
Middle	1.43 (0.86 ~ 2.37)	0.171			1.12 (0.73 ~ 1.71)	0.595		
Lower	1.44 (0.82 ~ 2.52)	0.201			1.13 (0.70 ~ 1.82)	0.609		
MPR								
No	1.00 (Reference)		1.00 (Reference)		1.00 (Reference)		1.00 (Reference)	
Yes	0.28 (0.19 ~ 0.42)	<.001	0.43 (0.26 ~ 0.69)	<.001	0.38 (0.28 ~ 0.54)	<.001	0.54 (0.37 ~ 0.81)	0.003
ypT								
T 0-2	1.00 (Reference)		1.00 (Reference)		1.00 (Reference)		1.00 (Reference)	
T 3-4	2.34 (1.65 ~ 3.31)	<.001	1.25 (0.84 ~ 1.86)	0.278	1.84 (1.36 ~ 2.49)	<.001	1.05 (0.74 ~ 1.49)	0.778
ypN								
ypN-	1.00 (Reference)		1.00 (Reference)		1.00 (Reference)		1.00 (Reference)	
ypN+	2.77 (1.95 ~ 3.94)	<.001	1.91 (1.31 ~ 2.77)	<.001	2.76 (2.02 ~ 3.78)	<.001	2.11 (1.51 ~ 2.95)	<.001

H–L, high ALI/low TL (low-risk); H–H/L–L, high ALI/high TL or low ALI/low TL (intermediate-risk); L–H, low ALI/high TL (high-risk).

A similar gradient was observed for DFS: adjusted HRs were 1.64 (95% CI 1.17–2.29, P = 0.004) and 2.62 (95% CI 1.74–3.95, P < 0.001) for the intermediate- and high-risk groups, respectively. Collectively, these findings indicate that, independent of other covariates, patients with low ALI and high TL faced approximately a threefold higher risk of death or recurrence, whereas those with only one adverse feature had intermediate survival outcomes.

### Nomogram construction and validation

**Figure 2:**
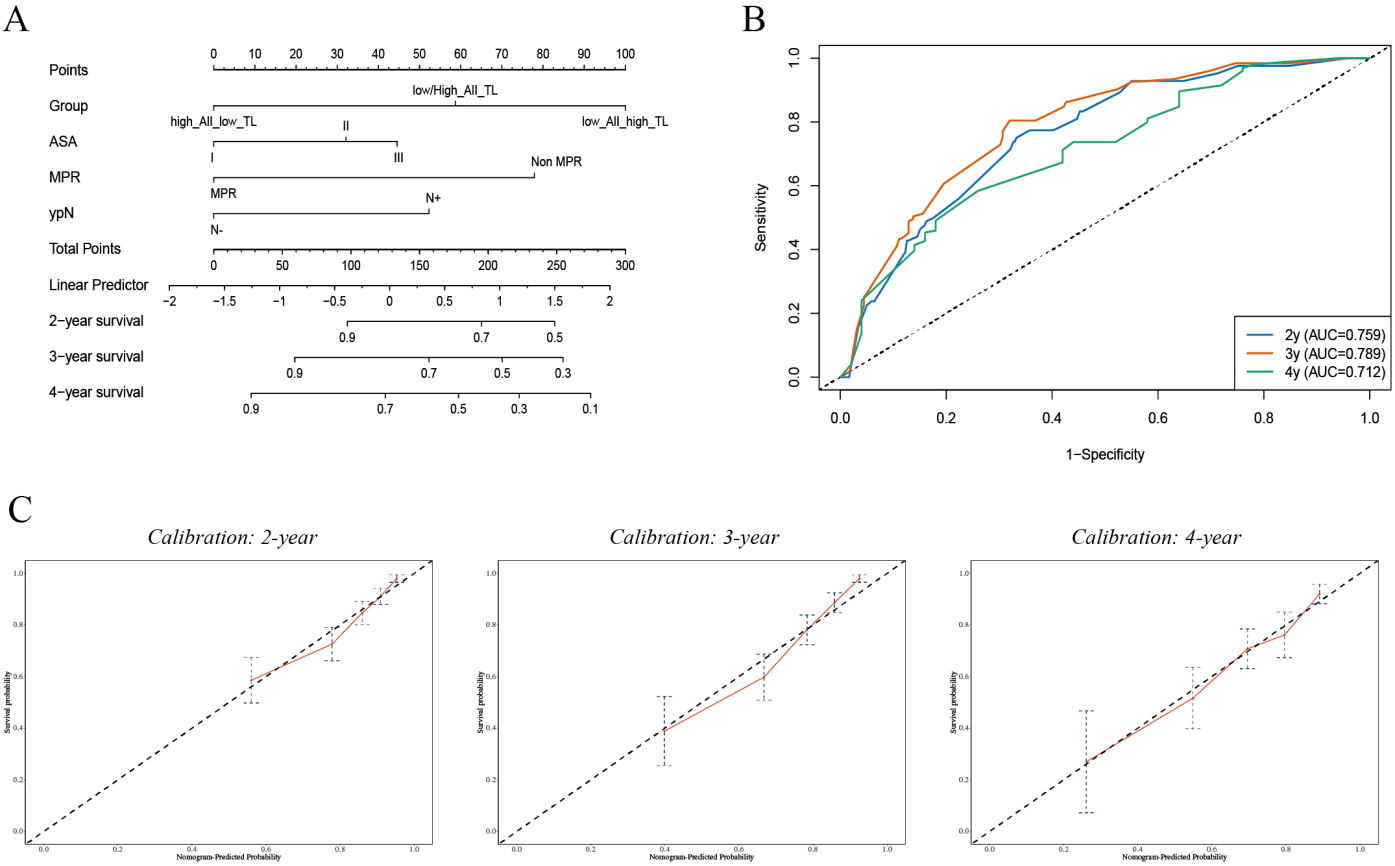
Development and validation of a prognostic model integrating ALI and TL. **(A)** Nomogram predicting 3-year overall survival based on ALI, TL, and clinicopathologic covariates. **(B)** Receiver operating characteristic (ROC) curve assessing discriminative performance of the model. **(C)** Calibration plot showing agreement between predicted and observed survival probabilities.

A prognostic nomogram predicting 2-, 3-, and 4-year OS was established based on the multivariable Cox model, incorporating ALI/TL risk group, ASA score, MPR status, and ypN stage (**Figure 2A**). Each variable was assigned a score proportional to its relative contribution to survival prediction, and total scores corresponded to estimated survival probabilities at each time point.

Calibration plots demonstrated excellent agreement between predicted and observed OS at 2, 3, and 4 years (**Figure 2B**), confirming the model’s robust calibration. The model exhibited strong discriminative ability, with a Harrell’s C-index of 0.742 (95% CI 0.668–0.816), indicating that in approximately 74% of comparable patient pairs, the model correctly identified the patient with the poorer prognosis. Time-dependent ROC analyses further supported its predictive accuracy, yielding AUCs of 0.759, 0.789, and 0.712 for 2-, 3-, and 4-year survival predictions, respectively (**Figure 2C**).

## Discussion

In this study, we demonstrated that combining ALI with TL provides powerful prognostic stratification in ESCC patients treated with neoadjuvant PD-1 inhibitor–based immunochemotherapy. To our knowledge, this is the first large-scale analysis evaluating ALI and TL in this emerging treatment setting. The key finding is that patients with a high ALI (≥31.22) and low TL (<0.224) had dramatically better survival and treatment responses than those with low ALI and high TL, with other patients falling in between. This three-tier risk stratification was independently associated with overall survival, disease-free survival, and the likelihood of achieving a major pathological remission. These results highlight the interplay between host inflammatory/nutritional status and tumor burden in determining outcomes of ESCC under immunochemotherapy, and suggest that both aspects should be considered when assessing patient prognosis.

Our findings regarding ALI are broadly consistent with prior reports in gastrointestinal malignancies while extending its applicability to the setting of immunochemotherapy (19, 26). Originally proposed as a composite prognostic index in advanced lung cancer, ALI has since demonstrated prognostic value across multiple tumor types, including gastric and esophageal cancers (26, 27). A low ALI thus signifies cachexia or malnutrition alongside an elevated NLR, which has been linked to aggressive tumor biology and immune evasion (28). In surgical series, ESCC patients with low ALI have shown worse survival and higher postoperative morbidity (27). Consistent with these data, our study confirmed ALI as a robust prognostic indicator: patients with high ALI experienced significantly longer OS and DFS than those with low ALI across all analyses.

Importantly, high ALI also correlated with increased pCR and MPR rates, suggesting enhanced tumor eradication following neoadjuvant therapy. One plausible mechanism is that well-nourished patients with lower systemic inflammation mount a more competent anti-tumor immune response (29, 30). A low NLR—an integral component of high ALI—reflects a predominance of lymphocytes relative to neutrophil-derived immunosuppressive factors, thereby potentially augmenting the activity of PD-1 inhibitors and cytotoxic agents (31, 32). Conversely, chronic inflammation and nutritional depletion in low-ALI patients may compromise T-cell function and tissue recovery, diminishing treatment efficacy. Clinically, ALI may thus serve as a readily available biomarker for identifying patients who could benefit from early nutritional optimization or anti-inflammatory interventions prior to initiating therapy.

Similarly, our results validate the prognostic relevance of circulating tumor markers in ESCC and introduce TL as a practical composite indicator of tumor burden. Elevated pre-treatment levels of CEA, SCC-Ag, and CA19–9 have each been linked to worse outcomes in esophageal cancer (22, 23). For example, Hu et al. reported that CEA > 2.28 ng/mL or SCC-Ag > 0.75 ng/mL was associated with significantly shorter OS and DFS after esophagectomy (23). These observations align with our univariate results showing adverse survival for patients with high individual markers. Using LASSO regression, we integrated the three markers into a single continuous variable—TL—which likely enhances stability and generalizability compared with any single marker. The weighting coefficients in the TL model suggest that CEA and SCC-Ag contribute most strongly, with CA19–9 providing additive value, consistent with previous evidence of their relative prognostic importance in ESCC. We observed that high TL (≥0.224) was independently associated with higher risks of recurrence and mortality.

From a biological perspective, patients with elevated TL likely harbor greater systemic tumor burden or occult micrometastatic disease, predisposing them to early relapse despite multimodal therapy (33). Notably, TL also correlated inversely with treatment response: TL-high patients had significantly lower pCR and MPR rates, indicating relative chemo-immunoresistance. This supports the intuitive notion that a heavier tumor load may be less amenable to eradication by a limited number of preoperative therapy cycles. Thus, TL provides a simple yet informative surrogate of tumor aggressiveness that can be derived from routine serologic testing. In our cohort, the statistically derived cut-off values (ALI < 31.22 and TL > 0.224) identify a distinct subgroup of patients facing a “double hit” of high tumor burden and compromised host defense. Clinically, an ALI below 31.22 likely represents a critical threshold where systemic inflammation and nutritional depletion significantly impair anti-tumor immunity, while a TL above 0.224 signifies an aggregate tumor burden that exceeds the host’s immune clearance capacity.

A novel contribution of this study lies in demonstrating the synergistic prognostic effect of combining ALI and TL. While each factor independently stratified risk, their integration yielded markedly superior discrimination. Patients with high ALI/low TL achieved excellent long-term outcomes (3-year OS = 84.7%; pCR = 21%), whereas those with low ALI/high TL exhibited poor treatment responses and dismal survival (3-year OS = 34.6%). Intermediate-risk patients displayed outcomes between these extremes, suggesting biologic interaction between host resilience and tumor aggressiveness. A robust host environment (high ALI) may partially counterbalance high tumor load through improved therapy tolerance and immune competence, whereas compromised host status (low ALI) may attenuate the benefit of a modest tumor burden. Taken together, these findings emphasize that prognosis in ESCC reflects the interplay between host immunity–nutrition and tumor biology, and that the combined ALI/TL model offers a more holistic and clinically interpretable representation of disease dynamics.

These insights have important implications for risk-adapted treatment strategies. Patients identified preoperatively as high-risk (low ALI/high TL) demonstrated minimal pathological response to standard immunochemotherapy, suggesting inherent resistance to this regimen. For this specific subgroup, “treatment intensification” should not imply abandoning surgery, but rather optimizing the induction strategy. This could primarily involve switching to nICRT to leverage the locoregional cytotoxic control of radiation, or enrolling in clinical trials testing novel agents. Additionally, aggressive nutritional and anti-inflammatory prehabilitation is warranted to reverse the compromised host status (low ALI) prior to surgery. In the postoperative setting, closer surveillance or adjuvant therapy may be warranted to mitigate recurrence risk (34). Conversely, low-risk patients (high ALI/low TL) achieved excellent outcomes with current protocols, suggesting that treatment de-escalation could be explored in future trials. Patients classified as intermediate-risk might continue to receive conventional regimens. Because both ALI and TL are derived from widely available clinical and laboratory data, they can be seamlessly incorporated into prognostic nomograms or decision-support algorithms, providing a pragmatic and low-cost alternative to more complex molecular or imaging biomarkers.

This study has several limitations. First, its retrospective design and recruitment from two centers may introduce selection bias and residual confounding. We minimized these effects through strict inclusion criteria and comprehensive multivariable adjustment, though external validation in independent prospective cohorts remains necessary. Second, the median follow-up of 42 months, while sufficient for early survival assessment, limits evaluation of long-term outcomes beyond 5 years. Third, as all patients received PD-1 inhibitor–based immunochemotherapy, our data do not allow direct comparison with chemotherapy-only regimens. Fourth, the Tumor Load Index was constructed and evaluated within the same cohort using LASSO Cox regression. Despite the use of cross-validation to minimize overfitting, the lack of an independent external validation set remains a limitation. Finally, the optimal cut-off values for ALI and TL identified in this study may be population-specific. Future multi-center studies are warranted to validate and refine these thresholds to ensure their generalizability across diverse clinical settings.

## Conclusion

In summary, this study demonstrates that pre-treatment ALI and TL synergistically predict prognosis in patients with ESCC treated with neoadjuvant immunochemotherapy followed by surgery. A high ALI, reflecting favorable nutritional and inflammatory status, together with a low TL, indicating minimal tumor burden, was associated with markedly improved survival and higher pathologic response rates. Future prospective studies and interventional trials are warranted to validate these biomarkers and explore strategies that modulate inflammation and tumor load to improve outcomes among high-risk (low ALI/high TL) ESCC patients.

## Data Availability

The original contributions presented in the study are included in the article/**Supplementary Material**. Further inquiries can be directed to the corresponding authors.

## Supplementary material

Supplementary Table 1 Univariable analyses of ALI, TL, and their combined classification for overall survival and disease-free survival.

Supplementary Figure 1 Overall survival (A) and disease-free survival (B) in the entire cohort of ESCC patients treated with neoadjuvant immunochemotherapy followed by surgery.

Supplementary Figure 2 Comparison of predictive performance among different prognostic models using Receiver Operating Characteristic (ROC) analysis.
