# Supplementary material for: Prognostic impact of advanced lung cancer inflammation index and tumor load index in esophageal squamous cell carcinoma after neoadjuvant immunochemotherapy
Source: Front Immunol. 2026 Jan 28;17:1724061. doi: 10.3389/fimmu.2026.1724061 (PMC12891139; doi:10.3389/fimmu.2026.1724061)
Supplement: Supplementary Table 1 — Univariable analyses of ALI, TL, and their combined classification for overall survival and disease-free survival. [file Table1.docx]

Supplementary Table 1 Univariable analyses of ALI, TL, and their combined classification for overall survival and disease-free survival.

|  |  | OS |  | | | DFS |  | |
| --- | --- | --- | --- | --- | --- | --- | --- | --- |
| Variables | *P* | HR (95%CI) | *P* | | | HR (95%CI) |  | |
|  |  |  |  |  |  |  |  |  |
| Tumor Load Group |  |  |  | | |  |  | |
| low |  | 1.00 (Reference) |  | | | 1.00 (Reference) |  | |
| high | <.001 | 2.12 (1.53 ~ 2.93) | <.001 | | | 1.76 (1.31 ~ 2.37) |  | |
| ALI group |  |  |  | | |  |  | |
| high |  | 1.00 (Reference) |  | | | 1.00 (Reference) |  | |
| low | <.001 | 2.98 (2.14 ~ 4.13) | <.001 | | | 2.37 (1.75 ~ 3.20) |  | |
| GROUP |  |  |  | | |  |  | |
| H–L |  | 1.00 (Reference) |  | | | 1.00 (Reference) |  | |
| H–H / L–L | <.001 | 2.19 (1.50 ~ 3.20) | 0.002 | | | 1.67 (1.20 ~ 2.33) |  | |
| L–H | <.001 | 5.05 (3.27 ~ 7.79) | | <.001 | 3.60 (2.43 ~ 5.33) | | |  |

Abbreviations: H–L, high ALI / low TL (low-risk); H–H / L–L, high ALI / high TL or low ALI / low TL (intermediate-risk); L–H, low ALI / high TL (high-risk).
