# Supplementary material for: Prognostic impact of advanced lung cancer inflammation index and tumor load index in esophageal squamous cell carcinoma after neoadjuvant immunochemotherapy
Source: Front Immunol. 2026 Jan 28;17:1724061. doi: 10.3389/fimmu.2026.1724061 (PMC12891139; doi:10.3389/fimmu.2026.1724061)

Supplementary Figure 1 Overall survival (A) and disease-free survival (B) in the entire cohort of ESCC patients treated with neoadjuvant immunochemotherapy followed by surgery.


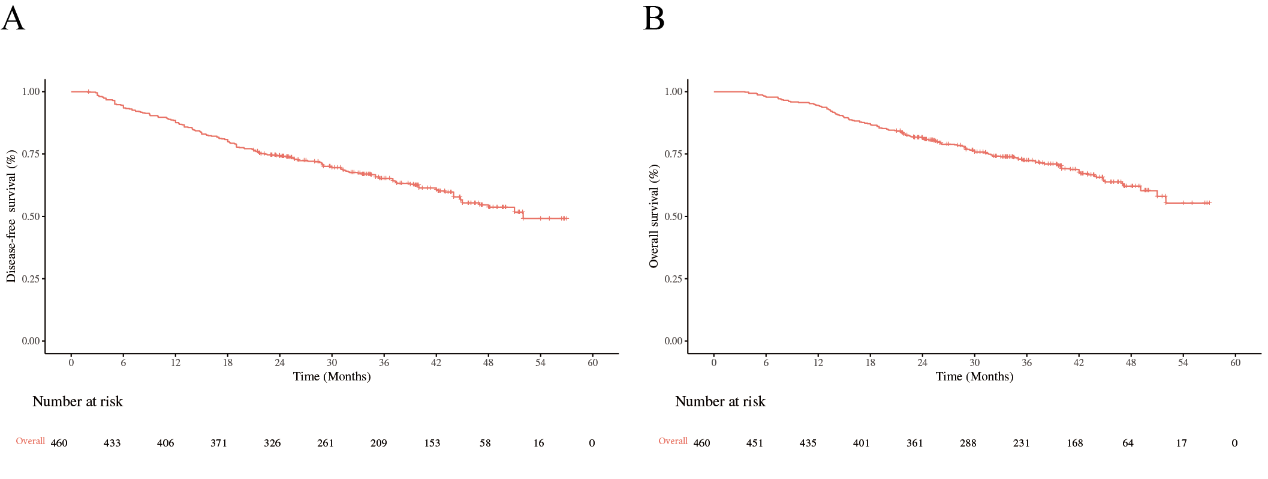


Supplementary Figure 2 Comparison of predictive performance among different prognostic models using Receiver Operating Characteristic (ROC) analysis.


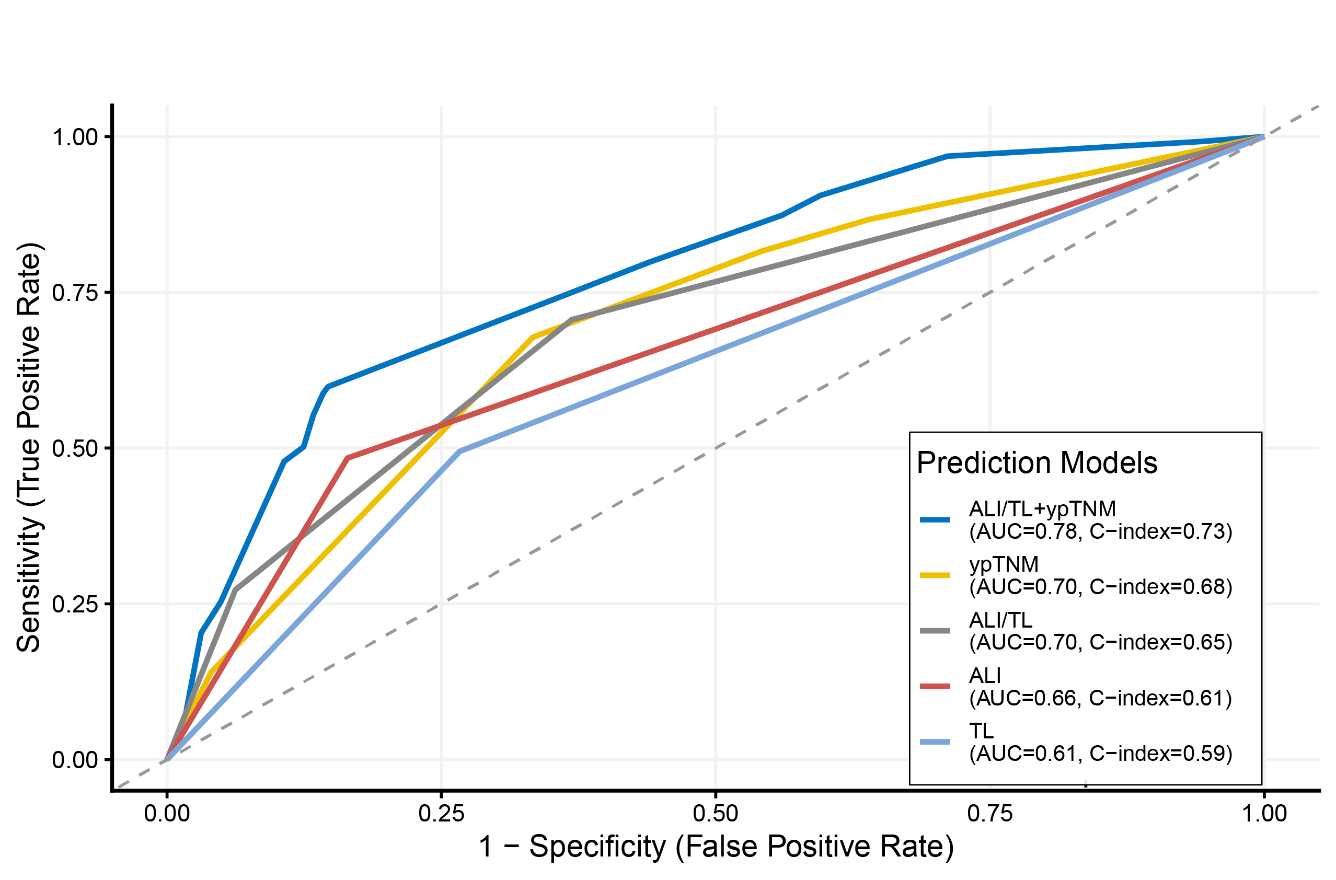

Supplement: Supplementary Figure 1 — Overall survival (A) and disease-free survival (B) in the entire cohort of ESCC patients treated with neoadjuvant immunochemotherapy followed by surgery. [file Table2.docx]
